# Association between any underlying health condition and COVID-19-associated hospitalization by age group, Washington State, 2020–2021: a retrospective cohort study

**DOI:** 10.1186/s12879-023-08146-7

**Published:** 2023-03-30

**Authors:** Kate H. McConnell, Anjum Hajat, Coralynn Sack, Stephen J. Mooney, Christine M. Khosropour

**Affiliations:** 1 Department of Epidemiology, University of Washington, Seattle, WA USA; 2 Department of Medicine, University of Washington, Seattle, WA USA; 3 Department of Environmental and Occupational Health Sciences, University of Washington, Seattle, WA USA

**Keywords:** Pandemics, Communicable diseases, Coronavirus, SARS-CoV-2, COVID-19, Comorbidity, Age factors, Electronic health records, Regression analysis, Washington

## Abstract

**Background:**

Presence of at least one underlying health condition (UHC) is positively associated with severe COVID-19, but there is limited research examining this association by age group, particularly among young adults.

**Methods:**

We examined age-stratified associations between any UHC and COVID-19-associated hospitalization using a retrospective cohort study of electronic health record data from the University of Washington Medicine healthcare system for adult patients with a positive SARS-CoV-2 test from February 29, 2020, to March 13, 2021. Any UHC was defined as documented diagnosis of at least one UHC identified by the CDC as a potential risk factor for severe COVID-19. Adjusting for sex, age, race and ethnicity, and health insurance, we estimated risk ratios (aRRs) and risk differences (aRDs), overall and by age group (18–39, 40–64, and 65 + years).

**Results:**

Among patients aged 18–39 (*N* = 3,249), 40–64 (*N* = 2,840), 65 + years (*N* = 1,363), and overall (*N* = 7,452), 57.5%, 79.4%, 89.4%, and 71.7% had at least one UHC, respectively. Overall, 4.4% of patients experienced COVID-19-associated hospitalization. For all age groups, the risk of COVID-19-associated hospitalization was greater for patients with any UHC vs. those without (18–39: 2.2% vs. 0.4%; 40–64: 5.6% vs. 0.3%; 65 + : 12.2% vs. 2.8%; overall: 5.9% vs. 0.6%). The aRR comparing patients with vs. those without UHCs was notably higher for patients aged 40–64 years (aRR [95% CI] for 18–39: 4.3 [1.8, 10.0]; 40–64: 12.9 [3.2, 52.5]; 65 + : 3.1 [1.2, 8.2]; overall: 5.3 [3.0, 9.6]). The aRDs increased across age groups (aRD [95% CI] per 1,000 SARS-CoV-2-positive persons for 18–39: 10 [2, 18]; 40–64: 43 [33, 54]; 65 + : 84 [51, 116]; overall: 28 [21, 35]).

**Conclusions:**

Individuals with UHCs are at significantly increased risk of COVID-19-associated hospitalization regardless of age. Our findings support the prevention of severe COVID-19 in adults with UHCs in all age groups and in older adults aged 65 + years as ongoing local public health priorities.

**Supplementary Information:**

The online version contains supplementary material available at 10.1186/s12879-023-08146-7.

## Background

Since its recognition in December 2019, coronavirus disease 2019 (COVID-19) – the disease caused by the coronavirus severe acute respiratory syndrome coronavirus 2 (SARS-CoV-2) – has had an unprecedented impact around the world [1]. As of September 2022, the COVID-19 pandemic has led to over 613 million confirmed cases and over 6 million confirmed deaths globally [1].

While most people with COVID-19 experience mild or moderate illness, some individuals develop severe outcomes (including hypoxia, respiratory failure, and multiorgan system dysfunction) and require hospitalization for management [2]. Adults aged 65 years and older and people with certain underlying health conditions (UHCs), such as cerebrovascular disease, chronic kidney disease, heart conditions, and diabetes mellitus, are at higher risk of developing severe COVID-19 [3–19]. However, even healthy young adults may suffer severe or life-threatening COVID-19 symptoms [20].

In the United States (US), COVID-19 disease patterns have differed by age group [21–25]. Young adults under age 40 have represented a substantial proportion of COVID-19 hospitalizations [26] and have had the highest incidence of COVID-19 cases during much of the pandemic [21]. Despite evidence demonstrating young adults are susceptible to severe COVID-19 and the high incidence of COVID-19 cases among young adults, there are relatively few studies exploring the relationship between UHCs and COVID-19 disease severity by age group [12–18]. Improved understanding of these age-stratified relationships – particularly among young adults – may inform public health action including risk communication and prioritization of vaccination and anti-SARS-CoV-2 therapy.

Mirroring national trends, young adults 40 years and under in Washington (WA) State have made up a notable percentage of COVID-19 hospitalizations [27] and have had the highest incidence of COVID-19 cases throughout much of the pandemic [28]. The aim of our study was to examine the association between any UHC and COVID-19-associated hospitalization, overall and stratified by age group, among patients in a large academic medical system in western WA State.

## Methods

### Study design, data source, setting, and population

We conducted a retrospective cohort study using electronic health record (EHR) data from the University of Washington Medicine (UWM) healthcare system – the largest academic medical system in the Central Puget Sound region. The Central Puget Sound region is in the western part of WA State and includes the Seattle metro area; it comprises 56% of the state’s population [29] and, as of September 2022, about 54% of its confirmed and probable COVID-19 cases [28].

Our study population included living adult patients engaged in care within UWM who tested positive for SARS-CoV-2 within UWM. We defined engagement in care within UWM as presence of at least one encounter (including telephone or telemedicine) in the UWM system from January 1, 2017 to February 28, 2020 (the day before SARS-CoV-2 testing began within UWM on February 29, 2020). We included patients who were aged 18 years or older as of February 28, 2020, who had at least one positive SARS-CoV-2 reverse transcription polymerase chain reaction (RT-PCR) test within UWM from February 29, 2020 to March 13, 2021 (14 days before the date of the data pull, to allow time for COVID-19-associated hospitalization following a positive test), and who were alive at the time of their first positive SARS-CoV-2 RT-PCR test. RT-PCR is the diagnostic standard for SARS-CoV-2 infection used within UWM.

### Data measures

Our cohort, as defined above, contained 7,456 adult UWM patients. Data were collected at the patient level. We defined any UHC (yes/no) as documented diagnosis of at least one UHC identified by the US Centers for Disease Control and Prevention (CDC) as a potential risk factor for severe COVID-19 (Supplementary Table 1 in Additional File 1) [19]. To identify documented UHCs, we evaluated EHR data from January 1, 2017 through the date of the first positive SARS-CoV-2 RT-PCR test. Most UHCs were defined by the presence of at least one relevant international classification of disease, tenth revision, clinical modification (ICD-10-CM) diagnosis code (Supplementary Table 1 in Additional File 1). In addition to ICD-10-CM data, EHR smoking status data were used to identify patients with the UHC of current or former smoking, and EHR height and weight data were used to calculate BMI (kg/m^2^) to identify patients with the UHCs of overweight and obesity. BMI was based on the most recent encounter from January 1, 2017 through the date of the first positive SARS-CoV-2 RT-PCR test for which height, weight, and the calculated BMI were biologically plausible. Biological plausibility for height was defined as ≥ 1.2 m and ≤ 2.4 m, for weight as ≥ 34.0 kg and ≤ 272.2 kg, and for BMI as ≤ 80.0 kg/m^2^ [30]. Plausible BMI data were recorded for 5,413 patients (72.6%). The median number of days between height and weight measurement and the first positive SARS-CoV-2 RT-PCR test was 358 (interquartile range = 601 days). Patients who had no indication of any UHCs as defined above were categorized as having no UHCs.

We defined COVID-19-associated hospitalization (yes/no) as an in-patient encounter within UWM that either (1) had an admit date at most three days prior to the date of a positive SARS-CoV-2 test and an indication in the admit note that the hospital encounter was associated with COVID-19, or (2) was within 14 days following a positive SARS-CoV-2 test; this is the standard approach validated and used by the University of Washington Institute of Translational Health Sciences to identify COVID-19-associated hospitalizations.

We also collected EHR data on potential confounders including age (in years) at the time of first positive SARS-CoV-2 RT-PCR test, sex assigned at birth (female/male), race and ethnicity (Hispanic or Latine, non-Hispanic American Indian or Alaska Native, non-Hispanic Asian, non-Hispanic Black, non-Hispanic Native Hawaiian or Pacific Islander, non-Hispanic White, not recorded), and health insurance status (public, private, uninsured, not recorded) as a measure of individual-level socioeconomic position. Four patients (< 0.1%) had implausible ages (≥ 120 years) or were missing data on sex assigned at birth and were dropped from the analysis, for a final analytic population of 7,452 patients. Missing race and ethnicity and health insurance data were classified as “not recorded.”

### Data analyses

We estimated adjusted risk ratios (aRRs) and risk differences (aRDs) of COVID-19-associated hospitalization (yes/no) by presence of any UHC (yes/no) using multivariable log-binomial and generalized linear regression with a Gaussian distribution and identity link function, respectively, both with Huber-White estimates of the standard error [31]. We adjusted for continuous age, sex assigned at birth, race and ethnicity, and health insurance status. We presented our results overall and stratified by age groups 18–39, 40–64, and 65 + years, which are consistent with the CDC’s reporting of age-stratified COVID-19 data [21]. Model 1 describes the model used to estimate the aRR in the total study population, and Model 2 describes the model used to estimate age-stratified aRRs; these models are comparable in structure to those used to estimate aRDs. Finally, we tested for interaction by age group of the association between any UHC and COVID-19-associated hospitalization using analysis of variance to compare nested models (e.g., in the case of the aRR, we compared Model 1 and Model 2). All analyses were performed in R version 4.1.2 (Vienna, Austria) [32].

$$\mathrm{log}\left({p}_{{hospitalization}_{i}}\right)={\beta }_{0}+{\beta }_{1}{X}_{{any UHC}_{i}}+{\beta }_{2}{X}_{{age group 40-64}_{i}}+{\beta }_{3}{X}_{{age group 65+}_{i}}+{\beta }_{j}{X}_{{confounder}_{{j}_{i}}}+\dots +{\beta }_{k}{X}_{{confounder}_{{k}_{i}}}$$

Model 1

$$\mathrm{log}\left({p}_{{hospitalization}_{i}}\right)={\beta }_{0}+{\beta }_{1}{X}_{{any UHC}_{i}}+{\beta }_{2}{X}_{{age group 40-64}_{i}}+{\beta }_{3}{X}_{{age group 65+}_{i}}+{\beta }_{j}{X}_{{confounder}_{{j}_{i}}}+\dots +{\beta }_{k}{X}_{{confounder}_{{k}_{i}}}+{\gamma }_{1}{X}_{{any UHC}_{i}}{X}_{{age group 40-64}_{i}}+{\gamma }_{2}{X}_{{any UHC}_{i}}{X}_{{age group 65+}_{i}}$$

Model 2

### Sensitivity analyses

We conducted three sensitivity analyses. First, to account for changes in vaccine availability, we limited the dataset to patients whose first positive SARS-CoV-2 RT-PCR test was before December 11, 2020, when the Food and Drug Administration approved the first COVID-19 vaccine under emergency use authorization [33]. Second, to account for local changes in circulating viral strains [34] and testing practices [35], we limited the dataset to patients whose first positive SARS-CoV-2 RT-PCR test was between July 1, 2020 and October 31, 2020, when we expect the dominant viral strain [36, 37] and testing practices [28, 38–41] were relatively stable in the Central Puget Sound region. Finally, since UHCs have different levels of evidence (conclusive, suggestive, and inconclusive) for their association with severe COVID-19 according to the CDC, we repeated the primary regression analyses with a narrower definition of any UHC to include only conditions for which there is a published meta-analysis or systematic review demonstrating a conclusive increase in risk of severe COVID-19 (Supplementary Table 1 in Additional File 1) [19].

## Results

Among 7,452 individuals, the average age was 45.7 years, 49.8% of patients were assigned female sex at birth, 43.9% were recorded as non-Hispanic White, 36.4% were on public health insurance, and 6.9% were uninsured (Table 1). Patients aged 18–39 years comprised the largest group in our study population (*N* = 3,249), followed by 40–64-year-olds (*N* = 2,840), and then those aged 65 + years (*N* = 1,363). The distribution of sex assigned at birth was similar for all age groups. Patients recorded as non-Hispanic White comprised a higher proportion of subjects aged 65 + (59.1%) compared to those aged 18–39 (41.5%) and 40–64 years (39.2%). The proportion of patients on public health insurance increased across age groups, and patients aged 40–64 years were more likely to be uninsured compared to patients aged 18–39 and 65 + years.

**Table 1:** Characteristics of patients with a positive SARS-CoV-2 RT-PCR test, by age group*

	**Total** **(*N* = 7,452)**		**18–39 years**^**†**^ **(*N* = 3**,**249)**		**40–64 years**^**†**^ **(*N* = 2,840)**		**65 + years**^**†**^ **(*N*** ** = 1,363)**	
**Characteristic**	***n***	**%**	***n***	**%**	***n***	**%**	***n***	**%**
Age (years)^†^ (mean ± SD)	45.7	± 19.1	27.9	± 6.2	51.8	± 7.1	75.5	± 8.3
Sex^‡^								
Female	3,712	49.8	1,694	52.1	1,346	47.4	672	49.3
Male	3,740	50.2	1,555	47.9	1,494	52.6	691	50.7
Race and ethnicity								
Hispanic or Latine	1,301	17.5	608	18.7	589	20.7	104	7.6
NH AIAN	70	0.9	30	0.9	27	1.0	13	1.0
NH Asian	635	8.5	284	8.7	204	7.2	147	10.8
NH Black	1,012	13.6	431	13.3	460	16.2	121	8.9
NH NHPI	147	2.0	64	2.0	69	2.4	14	1.0
NH White	3,268	43.9	1,349	41.5	1,113	39.2	806	59.1
Not recorded	1,019	13.7	483	14.9	378	13.3	158	11.6
Insurance^§^								
Private	3,934	52.8	1,933	59.5	1,375	48.4	626	45.9
Public	2,716	36.4	1,003	30.9	1,042	36.7	671	49.2
Uninsured	517	6.9	165	5.1	319	11.2	33	2.4
Not recorded	285	3.8	148	4.6	104	3.7	33	2.4
Any UHC^‖^								
No	2,111	28.3	1,380	42.5	586	20.6	145	10.6
Yes	5,341	71.7	1,869	57.5	2,254	79.4	1,218	89.4

*SARS-CoV-2*   Severe acute respiratory syndrome coronavirus 2, *RT-PCR*   Reverse transcription polymerase chain reaction, *SD*   Standard deviation, *NH*  Non-Hispanic, *AIAN*   American Indian or Alaska Native, *NHPI*   Native Hawaiian or Pacific Islander, *UHC*   Underlying health condition

^*^University of Washington Medicine healthcare system, 02/29/2020 to 03/13/2021

^†^Age at first positive SARS-CoV-2 RT-PCR test within University of Washington Medicine; 2 patients (< 0.1%) had implausible ages (≥ 120 years) and were dropped from the analysis

^‡^Sex assigned at birth; 2 patients (< 0.1%) were missing data on sex assigned at birth and dropped from the analysis

^§^A measure of individual-level socioeconomic position; to capture information about the pre-pandemic period, health insurance data were based on the most recent encounter from 01/01/2017 to 02/28/2020 for which health insurance data were available; private includes patients with Medicare Advantage (Part C), Medicare Supplement (Medigap), Medicare (Part D), employer-sponsored and individual plans, private military insurance, and other forms of private insurance; public includes patients on Medicaid, Medicare (Part A and/or B only), public military insurance, and other forms of public insurance; uninsured includes patients without health insurance and patients with Indian Health Services insurance [42, 43]

^‖^Conditions possibly associated with increased risk of severe coronavirus disease 2019 per the United States Centers for Disease Control and Prevention [19], (all yes/no) based on electronic health record data; see Supplementary Table 1 (in Additional File 1) for list of conditions

Overall, 71.7% of patients had at least one UHC, and this proportion increased across age groups (57.5%, 79.4%, and 89.4%, respectively) (Table 1). The distributions of population characteristics stratified by the presence of any UHC are given in Table 2. Of note, patients with any UHC (*N* = 5,341) compared to patients with no UHCs (*N* = 2,111) had a higher proportion of individuals assigned male sex at birth (53.1% vs. 42.8%), individuals recorded as non-Hispanic White (45.0% vs. 40.9%), and individuals on public health insurance (41.0% vs. 24.9%). The distributions of specific UHCs overall and stratified by age group are given in Supplementary Table 2 (see Additional File 1). Overall, the most common UHCs were smoking (26.0%), obesity (26.0%), overweight (24.2%), and hypertension (23.2%). For most UHCs, the proportion of patients with a documented diagnosis increased across age groups, though this was not true for some UHCs including obesity, substance use disorders, and human immunodeficiency virus.

**Table 2:** Characteristics of patients with a positive SARS-CoV-2 RT-PCR test, by any UHC*

	**Total** **(*N* = 7,452)**		**Any UHC**^**†**^*** (N* = 5,341)**		**No UHC**^**†**^ **(*N* = 2,111)**	
**Characteristic**	***n***	**%**	***n***	**%**	***n***	**%**
Age (years)^‡^ (mean ± SD)	45.7	± 19.1	49.4	± 18.9	36.3	± 16.0
Age group (years)^‡^						
18–39	3,249	43.6	1,869	35.0	1,380	65.4
40–64	2,840	38.1	2,254	42.2	586	27.8
65 +	1,363	18.3	1,218	22.8	145	6.9
Sex^§^						
Female	3,712	49.8	2,504	46.9	1,208	57.2
Male	3,740	50.2	2,837	53.1	903	42.8
Race and ethnicity						
Hispanic or Latine	1,301	17.5	934	17.5	367	17.4
NH AIAN	70	0.9	63	1.2	7	0.3
NH Asian	635	8.5	431	8.1	204	9.7
NH Black	1,012	13.6	763	14.3	249	11.8
NH NHPI	147	2.0	122	2.3	25	1.2
NH White	3,268	43.9	2,405	45.0	863	40.9
Not recorded	1,019	13.7	623	11.7	396	18.8
Insurance^‖^						
Private	3,934	52.8	2,667	49.9	1,267	60.0
Public	2,716	36.4	2,190	41.0	526	24.9
Uninsured	517	6.9	383	7.2	134	6.3
Not recorded	285	3.8	101	1.9	184	8.7

*SARS-CoV-2*   Severe acute respiratory syndrome coronavirus 2, *RT-PCR*   Reverse transcription polymerase chain reaction, *UHC*   Underlying health condition, *SD*   Standard deviation, *NH*   Non-Hispanic, *AIAN*   American Indian or Alaska Native, *NHPI*   Native Hawaiian or Pacific Islander

^*^University of Washington Medicine healthcare system, 02/29/2020 to 03/13/2021

^†^Conditions possibly associated with increased risk of severe coronavirus disease 2019 per the United States Centers for Disease Control and Prevention [19], (all yes/no) based on electronic health record data; see Supplementary Table 1 (in Additional File 1) for list of conditions

^‡^Age at first positive SARS-CoV-2 RT-PCR test within University of Washington Medicine; 2 patients (< 0.1%) had implausible ages (≥ 120 years) and were dropped from the analysis

^§^Sex assigned at birth; 2 patients (< 0.1%) were missing data on sex assigned at birth and dropped from the analysis

^‖^A measure of individual-level socioeconomic position; to capture information about the pre-pandemic period, health insurance data were based on the most recent encounter from 01/01/2017 to 02/28/2020 for which health insurance data were available; private includes patients with Medicare Advantage (Part C), Medicare Supplement (Medigap), Medicare (Part D), employer-sponsored and individual plans, private military insurance, and other forms of private insurance; public includes patients on Medicaid, Medicare (Part A and/or B only), public military insurance, and other forms of public insurance; uninsured includes patients without health insurance and patients with Indian Health Services insurance [42, 43]

### Risk of COVID-19-associated hospitalization by any underlying health condition and age group

Overall, 329 (4.4%) of the 7,452 patients with a positive SARS-CoV-2 RT-PCR test experienced COVID-19-associated hospitalization (Supplementary Table 3 in Additional File 1). Among patients with any UHC, 5.9% experienced COVID-19-associated hospitalization compared to just 0.6% of patients with no UHCs (crude RR [95% CI] = 10.4 [5.9, 18.5]). For all age groups, the proportion of patients hospitalized with COVID-19 was higher among those with any UHC compared to those with no UHCs.

**Fig. 1:**
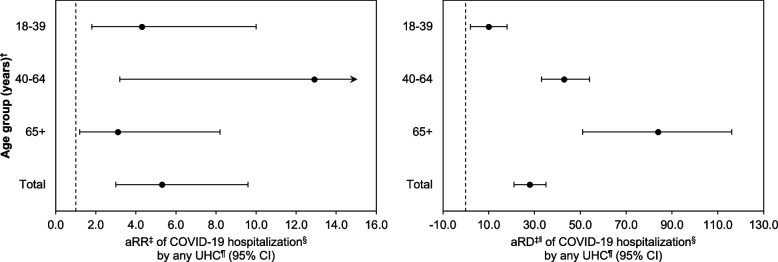
Adjusted risk ratios and differences of COVID-19-associated hospitalization by any UHC, stratified by age group. *COVID-19 = coronavirus disease 2019, UHC = underlying health condition, aRR = adjusted risk ratio, aRD = adjusted risk difference, CI = confidence interval. aRRs estimated by log-binomial regression using Huber-White estimates of the standard error; aRDs estimated using a generalized linear model with a Gaussian distribution and identity link function, and using Huber-White estimates of the standard error. Vertical dashed lines represent the null value of 1.0 in the aRR plot, and the null value of 0.0 in the aRD plot. Analysis of variance used to test for interaction by age group (aRR: *p*-value = 0.2; aRD: *p*-value < 0.001). *Among patients with a positive severe acute respiratory syndrome coronavirus 2 (SARS-CoV-2) reverse transcription polymerase chain reaction (RT-PCR) test, University of Washington Medicine healthcare system, 02/29/2020 to 03/13/2021. ^†^Age at first positive SARS-CoV-2 RT-PCR test within University of Washington Medicine. ^‡^Adjusted for continuous age (years), sex assigned at birth (female/male), race and ethnicity (Hispanic or Latine, non-Hispanic American Indian or Alaska Native, non-Hispanic Asian, non-Hispanic Black, non-Hispanic Native Hawaiian or Pacific Islander, non-Hispanic White, not recorded), and health insurance status (public, private, uninsured, not recorded); total models also adjusted for age group (18–39, 40–64, 65 + years). ^§^An indicator variable of COVID-19-associated hospitalization within University of Washington Medicine. ^‖^Difference in cumulative incidence per 1,000 SARS-CoV-2-positive persons over the 13-month study period. ^¶^Conditions possibly associated with increased risk of severe COVID-19 per the United States Centers for Disease Control and Prevention [19], (all yes/no) based on electronic health record data; see Supplementary Table 1 (in Additional File 1) for list of conditions

After adjustment for confounders, patients with any UHC had a 5.3-fold higher risk of COVID-19-associated hospitalization compared to those with no UHCs (aRR [95% CI] = 5.3 [3.0, 9.6]) (Fig. 1, Supplementary Table 3 in Additional File 1). Likewise, within each age group and on the relative scale, the risk of COVID-19-associated hospitalization was significantly higher among patients with any UHC compared to those with no UHCs. There was no statistical evidence of interaction by age group for the aRR (*p* = 0.2).

Overall, patients with any UHC had 28 additional COVID-19-associated hospitalizations per 1,000 SARS-CoV-2-positive persons during the 13-month study period compared to patients with no UHCs (aRD [95% CI] = 28 [21, 35]) (Fig. 1, Supplementary Table 3 in Additional File 1). Similarly, within each age group and on the absolute scale, the risk of COVID-19-associated hospitalization was significantly higher among patients with any UHC compared to those with no UHCs. There was statistical evidence of interaction by age group for the aRD (*p* < 0.001).

### Sensitivity analyses

The results of all three sensitivity analyses were consistent with those of the primary analyses (Supplementary Tables 4–6 in Additional File 1). Of note, using a narrower definition of any UHC yielded statistical evidence of interaction by age group for the aRR (*p* = 0.04) (Supplementary Table 6 in Additional File 1).

## Discussion

In this retrospective cohort study leveraging EHR data from a large academic medical center, we found that 4.4% of the 7,452 living adult patients engaged in care within UWM who tested positive for SARS-CoV-2 within UWM experienced COVID-19-associated hospitalization. Overall, patients with documented UHCs had a 5.3-fold higher risk of COVID-19-associated hospitalization compared to those without UHCs, and this positive relative association persisted across age groups. In addition, overall, patients with any UHC had 28 additional COVID-19-associated hospitalizations per 1,000 SARS-CoV-2-positive persons over the 13-month study period compared to those without UHCs, and the magnitude of this absolute association differed significantly across age groups, with older groups experiencing larger absolute differences in risk. Our findings suggest people with UHCs of all ages – including young and middle-aged adults < 65 years – are at elevated risk of COVID-19-associated hospitalization on both relative and absolute scales, and that the absolute difference in risk of COVID-19-associated hospitalization between those with and without UHCs increases with age.

Previous studies of the association between UHCs and severe COVID-19 defined severe outcomes in several ways, including clinical symptoms, hospitalization, intensive care unit admission, and mortality [4–18]. Most studies examined individual UHCs, rather than a composite measure of any UHC. A priori, we intended to explore age-stratified associations between COVID-19-associated hospitalization and individual UHCs in addition to our composite measure of any UHC, but ultimately did not due to small sample size. In addition, most previous studies calculated only relative measures of excess risk, rather than both relative and absolute measures as we did here. From an epidemiologic methods standpoint, our findings illustrate the value of presenting both relative and absolute measures of excess risk, which provide different perspectives and together offer a more complete understanding of a risk factor and its potential public health impact [44]. Our estimated overall aRR of 5.3 is consistent with the broad range of other published estimates of relative measures of excess risk [6–12]. For example, estimated hazard ratios for the association between dementia and severe COVID-19 ranged from 1.4 to 7.7 (95% CI range = 1.2, 39.6), and odds ratios for the association between liver cirrhosis and severe COVID-19 from 3.2 to 5.9 (95% CI range = 0.9, 27.7) in one review [11]. In addition, limited previous studies of the association between UHCs and severe COVID-19 stratified by age group have found the relative risk of severe COVID-19 comparing those with and without UHCs is higher in younger than in older people [12–18]. Although we did not find statistical evidence of effect modification by age group for the aRR in our primary analysis, the magnitudes of the estimated effects and the results of our sensitivity analysis using a narrower definition of any UHC, which did yield statistical evidence of interaction by age group for the aRR, are consistent with these previous findings.

Several of our findings are consistent with clinical expectations based on age as a risk factor for disease [45]. We found that the risk of COVID-19-associated hospitalization among those with and without UHCs increased with age, and that the aRDs describing the association between any UHC and COVID-19-associated hospitalization increased across age groups. A more surprising finding is that – although we did not find statistical evidence of interaction by age group and estimated 95% CIs were wide – qualitatively, the magnitude of the estimated aRR of COVID-19-associated hospitalization was higher among 18–39-year-olds (aRR = 4.3) and especially 40–64-year-olds (aRR = 12.9) compared to those aged 65 + years (aRR = 3.1). In addition, using a narrower definition of any UHC resulted in improved precision and statistical evidence of interaction by age group for the aRR. These findings suggest the presence of any UHC may be a stronger risk factor for COVID-19-associated hospitalization in younger adults and especially middle-aged adults compared to older adults, which is consistent with previous research of severe COVID-19 outcomes [12–18]. One possible explanation is that individuals in our study aged 65 + years with UHCs were healthy enough to survive to at least 65 years of age; thus, they may have been somewhat resilient against COVID-19, and, on the relative scale, their risk of COVID-19-associated hospitalization compared to their same-age counterparts without UHCs was not as different as it was for the other two age groups. On the other hand, the group of 40–64-year-olds with UHCs in our study likely comprised individuals in poor health who may not have survived to age 65; thus, they were less resilient against COVID-19 and their relative risk of a severe outcome was notably different from other middle-aged adults without UHCs.

At present in September 2022, adults of all ages with UHCs and older adults are priority populations for COVID-19 vaccination, including boosters, in WA State [46, 47], and for the use of anti-SARS-CoV-2 therapy among non-hospitalized adults with COVID-19 [48]. Our findings support the prevention of COVID-19 through vaccination and the prevention of progression to severe disease through anti-SARS-CoV-2 treatment in adults with UHCs in all age groups – including young and middle-aged adults < 65 years of age – and in adults aged 65 + years as ongoing local public health priorities.

We observed differences in our estimated relative and absolute measures of excess risk by age group, suggesting age-group-specific COVID-19 morbidity and mortality prevention activities, including risk communication, may be an appropriate approach for local health departments. Effective risk communication contributes to adequate understanding of personal risk, which has community-level health consequences [49], and previous studies show age-group-specific risk communication may be advantageous in the context of other health phenomena [50, 51] and in the context of COVID-19 [52–54]. Media reports and government messaging about COVID-19 – especially early in the pandemic – focused primarily on threats to older individuals, raising concerns that younger people might erroneously assume they were less likely to contract SARS-CoV-2 and/or they were invulnerable to severe COVID-19 outcomes [49, 52]. While older adults are at higher risk of severe COVID-19 compared to younger adults [3], even healthy young adults may develop severe or fatal COVID-19 symptoms [20]. In our study, we found that – while older adults with and without UHCs appeared to be at higher risk of COVID-19-associated hospitalization compared to younger adults – on the relative scale, young adults aged 18–39 years and especially middle-aged adults aged 40–64 years with any UHC were at higher risk than older adults aged 65 + years with any UHC when compared to their same-age counterparts with no UHCs. These results support age-group-specific risk communication in the Central Puget Sound region. As discussed above, our use of a composite measure of any UHC sets our study apart from most previous research, and may be useful for simplified public health messaging.

Our study had several limitations. First, we analyzed EHR data, which are subject to data quality concerns including high levels of missingness for certain variables, specifically race, ethnicity, and health insurance status in our data. Since EHR data often are missing not at random [55, 56], we did not employ multiple imputation methods, which may further increase bias under missing not at random conditions [57]. Instead, we classified individuals missing these data as “not recorded.” Inclusion of these “not recorded” categories, which may be highly heterogenous, may have limited our ability to statistically control confounding and led to bias [58, 59]. We also assumed patients who were missing EHR data used for UHC identification (e.g., smoking status) did not have that UHC, which may have resulted in differential exposure misclassification if patients hospitalized with COVID-19 within UWM were more likely to regularly encounter the UWM system (or less likely to receive care at outside institutions, for which records may be missing from the UWM database) and, therefore, had more complete UWM medical records or more opportunities for UHC diagnosis; this could have falsely exaggerated or falsely minimized estimated associations. In addition, we used presence of at least one relevant ICD-10-CM code to define most UHCs, which also may have resulted in differential exposure misclassification if this definition was too broad (e.g., a single code reflected a diagnostic lab work order, rather than actual diagnosis of the UHC) [60]. Second, it was not feasible for us to determine indication for SARS-CoV-2 testing (e.g., SARS-CoV-2 exposure, COVID-19 symptoms, pre-operative testing, etc.), which may be associated with COVID-19-associated hospitalization. While we do not expect the distribution of indication for testing to have impacted our aRR and aRD estimates, it may have impacted the proportion of patients in our study population who were hospitalized with COVID-19. Third, we only captured COVID-19-associated hospitalizations if they occurred within the UWM system, which may have led to differential outcome misclassification if patients without UHCs (who may have been less likely to be regularly engaged in care within UWM), were more likely to be hospitalized with COVID-19 at another facility. Furthermore, differential outcome misclassification may have occurred if patients who tested positive for SARS-CoV-2 within the prior 14 days were hospitalized for other emergent reasons but never experienced severe COVID-19 symptoms, or if patients died from severe COVID-19 without being hospitalized. Fourth, our cohort only included people we identified as being regularly engaged in care within the UWM system and who had a positive SARS-CoV-2 RT-PCT test within UWM, which may have increased the likelihood of selection bias from the oversampling of individuals with UHCs at increased risk of COVID-19-associated hospitalization. Fifth, the number of patients in our study without UHCs was relatively small and those individuals experienced few COVID-19-associated hospitalizations, especially within age group strata, which resulted in statistical limitations including poor precision. Finally, we used data from a single academic medical system, so the generalizability of our findings is unknown.

## Conclusions

In conclusion, our findings support the prevention of severe COVID-19 (e.g., via vaccination, including boosters, and anti-SARS-CoV-2 therapy) in adults with UHCs in all age groups – including young and middle-aged adults < 65 years of age – and in older adults aged 65 + years as ongoing local public health priorities [46–48]. Moreover, our findings suggest age-group-specific risk communication may be an appropriate approach in the Central Puget Sound region. Although we are in a period of relaxed COVID-19 prevention strategies in the US [61], we believe our work highlights the importance of age-group-specific research and public health action in our current stage of the SARS-CoV-2 pandemic, potential future waves of this SARS-CoV-2 pandemic, and potential future epidemics and pandemics of other pathogens.

## Supplementary Information

**Additional file 1.**

## Data Availability

The datasets analyzed in this study are not publicly available as they contain protected health information. De-identified datasets are available from the corresponding author and with permission from the University of Washington Institute of Translational Health Sciences upon reasonable request.

## Abbreviations

UHC: Underlying health condition
(a) RR: (Adjusted) risk ratio
(a) RD: (Adjusted) risk difference
COVID-19: Coronavirus disease 2019
SARS-CoV-2: Severe acute respiratory syndrome coronavirus 2
US: United States
WA: Washington
EHR: Electronic health record
UWM: University of Washington Medicine
RT-PCR: Reverse transcription polymerase chain reaction
CDC: Centers for Disease Control and Prevention
ICD-10-CM: International classification of disease, tenth revision, clinical modification
