# Supplementary material for: Association between any underlying health condition and COVID-19-associated hospitalization by age group, Washington State, 2020–2021: a retrospective cohort study
Source: BMC Infect Dis. 2023 Mar 30;23:193. doi: 10.1186/s12879-023-08146-7 (PMC10062257; doi:10.1186/s12879-023-08146-7)
Supplement: Supplementary file 1 — Additional file 1. [file 12879_2023_8146_MOESM1_ESM.pdf]

SUPPLEMENTARY TABLES

Supplementary Table 1. UHCs possibly associated with increased risk of severe COVID-19 per the United States Centers for Disease Control and Prevention and identifying ICD-10-CM codes

**UHC***		**ICD-10-CM code†**	**ICD-10-CM description**
	Asthma	J45	Asthma
*	Bronchiectasis	J47	Bronchiectasis
	Bronchopulmonary dysplasia	P27.1	Bronchopulmonary dysplasia originating in the perinatal period
*	Cancer	C00-C96	Malignant neoplasms
*	Cerebrovascular disease	I60-I69	Cerebrovascular diseases
*	Chronic kidney disease	N18	Chronic kidney disease (CKD)
*	Chronic obstructive pulmonary disorder	J44	Other chronic obstructive pulmonary disease
*	Corticosteroids or other immunosuppressive medications	D84.821; Z79.52; Z92.25	Immunodeficiency due to drugs; Long term (current) use of systemic steroids; personal history of immunosuppression therapy
*	Cystic fibrosis	E84	Cystic fibrosis
*	Diabetes mellitus, type 1	E10	Type 1 diabetes mellitus
*	Diabetes mellitus, type 2	E11	Type 2 diabetes mellitus
*	Down syndrome	Q90	Down syndrome
*	Heart conditions (e.g., heart failure, coronary artery disease, cardiomyopathies)	I20-I25; I30-I52	Ischemic heart disease; Other forms of heart disease
*	Human immunodeficiency virus	B20; Z21	Human immunodeficiency virus (HIV) disease; Asymptomatic human immunodeficiency virus (HIV) infection status
	Hypertension	I10	Essential (primary) hypertension
*	Immune deficiencies	D80; D81; D82; D83; D84.0; D84.1; D84.81; D84.822; D84.89; D84.9	Immunodeficiency with predominantly antibody defects; Combined immunodeficiencies; Immunodeficiency associated with other major defects; Common variable immunodeficiency; Lymphocyte function antigen-1 [LFA-1] defect; Defects in the complement system; Immunodeficiency due to conditions classified elsewhere; Immunodeficiency due to external causes; Other immunodeficiencies; Immunodeficiency, unspecified
*	Liver disease, chronic (cirrhosis, non-alcoholic fatty liver disease, alcoholic liver disease, autoimmune hepatitis)	K70-K77	Diseases of liver
*	Lung disease, interstitial	J80-J84	Other respiratory diseases principally affecting the interstitium
*	Lung disease, other	I28; J41; J42; J43; J60-J70; J85-J86; J90-J94; Z99.81	Other diseases of pulmonary vessels; Simple and mucopurulent chronic bronchitis; Unspecified chronic bronchitis; Emphysema; Lung diseases due to external agents; Suppurative and necrotic conditions of the lower respiratory tract; Other diseases of the pleura; Dependence on supplemental oxygen
*	Mental health disorders (mood disorders, e.g., depression and schizophrenia spectrum disorders)	F20-F29; F30-F39	Schizophrenia, schizotypal, delusional, and other non-mood psychotic disorders; Mood (affective) disorders
*	Neurologic conditions	F01; F02; F03; G10-G14; G20; G30-G32; G35; G45; G70; G80-G83	Vascular dementia; Dementia in other diseases classified elsewhere; Unspecified dementia; Systemic atrophies primarily affecting the central nervous system; Parkinson's disease; Other degenerative diseases of the nervous system; Multiple sclerosis; Transient cerebral ischemic attacks and related syndromes; Myasthenia gravis and other myoneural disorders; Cerebral palsy and other paralytic syndromes
*	Obesity (BMI $\geq$30kg/m^2)‡	E66.0; E66.1; E66.2; E66.8; E66.9	Obesity due to excessive calories; Drug-induced obesity; Morbid (severe) obesity with alveolar hypoventilation; Other obesity; Obesity, unspecified

Supplementary Table 1. UHCs possibly associated with increased risk of severe COVID-19 per the United States Centers for Disease Control and Prevention and identifying ICD-10-CM codes

UHC*		ICD-10-CM code†	ICD-10-CM description
	Overweight (BMI 25≥29kg/m^2)‡	E66.3	Overweight
*	Pregnancy and recent pregnancy	Z33; Z34; Z36; Z37; Z38; Z39; Z3A	Pregnant state; Encounter for supervision of normal pregnancy; Encounter for antenatal screening of mother; Outcome of delivery; Liveborn infants according to place of birth and type of delivery; Encounter for maternal postpartum care and examination; Weeks of gestation
*	Pulmonary hypertension and pulmonary embolism	I26; I27	Pulmonary embolism; Other pulmonary heart disease
	Sickle cell disease	D57	Sickle-cell disorders
*	Smoking, current and former‡	F17; Z72.0; Z87.891	Nicotine dependence; Tobacco use; Personal history of nicotine dependence
*	Solid organ or blood stem cell transplantation	Z94; T86	Transplanted organ and tissue status; Complications of transplanted organs and tissue
	Substance use disorders	F10-F16; F18; F19	Alcohol related disorders; Opioid related disorders; Cannabis related disorders; Sedative, hypnotic, or anxiolytic related disorders; Cocaine related disorders; Other stimulant related disorders; Hallucinogen related disorders; Inhalant related disorders; Other psychoactive substance related disorders
	Thalassemia	D56	Thalassemia
*	Tuberculosis	A15	Respiratory tuberculosis

UHC=underlying health condition, ICD-10-CM=international classification of disease, tenth revision, clinical modification, COVID-19=coronavirus disease 2019, BMI=body mass index

*UHCs for which there is a published meta-analysis or systematic review demonstrating a conclusive increase in risk of severe COVID-19 according to the United States Centers for Disease Control and Prevention [1] are marked with an asterisk

†ICD-10-CM codes and other electronic health record (EHR) data for all underlying health conditions were evaluated from 01/01/2017 to the date of the first positive reverse transcription polymerase chain reaction (RT-PCR) test for severe acute respiratory syndrome coronavirus 2 (SARS-CoV-2), except those for pregnancy and recent pregnancy, which were evaluated for the 60 days prior to the date of the first positive RT-PCR test for SARS-CoV-2

‡In addition to the ICD-10-CM codes indicated, EHR height and weight data were used to calculate BMI to identify patients with overweight and obesity, and EHR smoking data were used to identify patients with a history of smoking. BMI was based on the most recent encounter between 01/01/2017 and the date of the first positive SARS-CoV-2 RT-PCR test for which height, weight, and the calculated BMI were biologically plausible. Biological plausibility for height was defined as ≥1.2m and ≤2.4m, for weight as ≥34.0kg and ≤272.2kg, and for BMI as ≤80.0kg/m^2 [2]. Patients who had a smoking status of current or former smoker recorded at any encounter between 01/01/2017 and the date of the first positive SARS-CoV-2 RT-PCR test were classified as current or former smokers in our analyses

Supplementary Table 2. Distribution of UHCs among patients with a positive SARS-CoV-2 RT-PCR test, by age group, University of Washington Medicine healthcare system, February 29, 2020-March 13, 2021

	**UHC***	**Total N = 7,452**		**18-39 years† N = 3,249**		**40-64 years† N = 2,840**		**65+ years† N = 1,363**	
		**n**	**%**	**n**	**%**	**n**	**%**	**n**	**%**
	None	2,111	28.3	1,380	42.5	586	20.6	145	10.6
	Asthma	499	6.7	181	5.6	205	7.2	113	8.3
*	Bronchiectasis	42	0.6	2	0.1	14	0.5	26	1.9
	Bronchopulmonary dysplasia	0	0.0	0	0.0	0	0.0	0	0.0
*	Cancer	412	5.5	48	1.5	156	5.5	208	15.3
*	Cerebrovascular disease	346	4.6	26	0.8	117	4.1	203	14.9
*	Chronic kidney disease	418	5.6	31	1.0	157	5.5	230	16.9
*	Chronic obstructive pulmonary disorder	229	3.1	2	0.1	89	3.1	138	10.1
*	Corticosteroids or other immunosuppressive medications	106	1.4	19	0.6	53	1.9	34	2.5
*	Cystic fibrosis	3	<0.1	2	0.1	1	<0.1	0	0.0
*	Diabetes mellitus, type 1	78	1.0	27	0.8	38	1.3	13	1.0
*	Diabetes mellitus, type 2	954	12.8	79	2.4	489	17.2	386	28.3
*	Down syndrome	12	0.2	3	0.1	9	0.3	0	0.0
*	Heart conditions	1,009	13.5	87	2.7	373	13.1	549	40.3
*	Human immunodeficiency virus	119	1.6	27	0.8	81	2.9	11	0.8
	Hypertension	1,729	23.2	131	4.0	837	29.5	761	55.8
*	Immune deficiencies	37	0.5	4	0.1	19	0.7	14	1.0
*	Liver disease, chronic	371	5.0	56	1.7	204	7.2	111	8.1
*	Lung disease, interstitial	182	2.4	12	0.4	86	3.0	84	8.2
*	Lung disease, other	425	5.7	44	1.4	175	6.2	206	15.1
*	Mental health disorders	1,217	16.3	389	12.0	514	18.1	314	23.0
*	Neurologic conditions	555	7.4	39	1.2	144	5.1	372	27.3
*	Obesity (BMI ≥30kg/m^2)	1,935	26.0	603	18.6	960	33.8	372	27.3
	Overweight (BMI 25≥29kg/m^2)	1,803	24.2	683	21.0	749	26.4	371	27.2
*	Pregnancy and recent pregnancy	49	0.7	48	1.5	1	<0.1	0	0.0
*	Pulmonary hypertension and pulmonary embolism	174	2.3	20	0.6	75	2.6	79	5.8
	Sickle cell disease	10	0.1	7	0.2	3	0.1	0	0.0
*	Smoking, current and former	1,937	26.0	564	17.4	859	30.2	514	37.7
*	Solid organ or blood stem cell transplantation	108	1.4	18	0.6	49	1.7	41	3.0
	Substance use disorders	609	8.2	209	6.4	307	10.8	93	6.8
	Thalassemia	10	0.1	5	0.2	5	0.2	0	0.0
*	Tuberculosis	16	0.2	5	0.2	7	0.2	4	0.3

UHC=underlying health condition, SARS-CoV-2=severe acute respiratory syndrome coronavirus 2, RT-PCR=reverse transcription polymerase chain reaction, BMI=body mass index
Columns may sum to greater than 100% due to overlapping health conditions
*Conditions possibly associated with increased risk of severe coronavirus disease 2019 (COVID-19) per the United States Centers for Disease Control and Prevention [1]; UHCs for which there is a published meta-analysis or systematic review demonstrating a conclusive increase in risk of severe COVID-19 are marked with an asterisk
†Age at first positive SARS-CoV-2 RT-PCR test within University of Washington Medicine

Supplementary Table 3. Risks, risk ratios, and risk differences of COVID-19-associated hospitalization by presence of any UHC among patients with a positive SARS-CoV-2 RT-PCR test, overall and by age group, University of Washington Medicine healthcare system, February 29, 2020-March 13, 2021

Age group (years)*	Any UHC†	Risk of COVID-19 hospitalization‡		Crude RR		Adjusted RR§		Crude RD‖		Adjusted RD§‖	
		n/N	%	RR	95% CI	aRR	95% CI	RD	95% CI	aRD	95% CI
18-39	Yes	42 / 1,869	2.2	5.2	2.2, 12.1	4.3	1.8, 10.0	18	11, 26	10	2, 18
	No	6 / 1,380	0.4	*Referent*		*Referent*		*Referent*		*Referent*	
40-64	Yes	126 / 2,254	5.6	16.4	4.1, 66.1	12.9	3.2, 52.5	52	42, 63	43	33, 54
	No	2 / 586	0.3	*Referent*		*Referent*		*Referent*		*Referent*	
65+	Yes	149 / 1,218	12.2	4.4	1.7, 11.8	3.1	1.2, 8.2	95	62, 127	84	51, 116
	No	4 / 145	2.8	*Referent*		*Referent*		*Referent*		*Referent*	
Total	Yes	317 / 5,341	5.9	10.4	5.9, 18.5	5.3	3.0, 9.6	54	47, 61	28	21, 35
	No	12 / 2,111	0.6	*Referent*		*Referent*		*Referent*		*Referent*	

COVID-19=coronavirus disease 2019, UHC=underlying health condition, SARS-CoV-2=severe acute respiratory syndrome coronavirus 2, RT-PCR=reverse transcription polymerase chain reaction, (a)RR=(adjusted) risk ratio, (a)RD=(adjusted) risk difference, CI=confidence interval
RRs estimated by log-binomial regression using Huber-White estimates of the standard error; RDs estimated using a generalized linear model with a Gaussian distribution and identity link function, and using Huber-White estimates of the standard error
Analysis of variance used to test for interaction by age group (aRR: p-value=0.2; aRD: p-value<0.001)
*Age at first positive SARS-CoV-2 RT-PCR test within University of Washington Medicine
†Conditions possibly associated with increased risk of severe COVID-19 per the United States Centers for Disease Control and Prevention [1], (all yes/no) based on electronic health record data; see Supplementary Table 1 for list of conditions
‡An indicator variable of COVID-19-associated hospitalization within University of Washington Medicine
§Adjusted for continuous age (years), sex assigned at birth (female/male), race and ethnicity (Hispanic or Latine, non-Hispanic American Indian or Alaska Native, non-Hispanic Asian, non-Hispanic Black, non-Hispanic Native Hawaiian or Pacific Islander, non-Hispanic White, not recorded), and health insurance status (public, private, uninsured, not recorded); total models also adjusted for age group (18-39, 40-64, 65+ years)
‖Difference in cumulative incidence per 1,000 SARS-CoV-2-positive persons over the 13-month study period

Supplementary Table 4. Risks, risk ratios, and risk differences of COVID-19-associated hospitalization by presence of any UHC among patients with a positive SARS-CoV-2 RT-PCR test, overall and by age group, University of Washington Medicine healthcare system, February 29, 2020-December 10, 2020

Age group (years)*	Any UHC†	Risk of COVID-19 hospitalization‡		Crude RR		Adjusted RR§		Crude RD‖		Adjusted RD§‖	
		n/N	%	RR	95% CI	aRR	95% CI	RD	95% CI	aRD	95% CI
18-39	Yes	39 / 1,602	2.4	5.1	2.2, 12.0	4.1	1.7, 9.7	20	11, 28	11	3, 20
	No	6 / 1,257	0.5	*Referent*		*Referent*		*Referent*		*Referent*	
40-64	Yes	100 / 1,825	5.5	27.1	3.8, 193.7	21.6	3.0, 155.2	53	42, 64	44	33, 56
	No	1 / 494	0.2	*Referent*		*Referent*		*Referent*		*Referent*	
65+	Yes	111 / 996	11.1	4.5	1.5, 14.1	3.4	1.1, 10.4	87	53, 121	77	43, 111
	No	3 / 122	2.5	*Referent*		*Referent*		*Referent*		*Referent*	
Total	Yes	250 / 4,423	5.7	10.6	5.6, 19.9	5.7	3.0, 10.7	51	44, 59	28	21, 35
	No	10 / 1,873	0.5	*Referent*		*Referent*		*Referent*		*Referent*	

COVID-19=coronavirus disease 2019, UHC=underlying health condition, SARS-CoV-2=severe acute respiratory syndrome coronavirus 2, RT-PCR=reverse transcription polymerase chain reaction, (a)RR=(adjusted) risk ratio, (a)RD=(adjusted) risk difference, CI=confidence interval
RRs estimated by log-binomial regression using Huber-White estimates of the standard error; RDs estimated using a generalized linear model with a Gaussian distribution and identity link function, and using Huber-White estimates of the standard error
Analysis of variance used to test for interaction by age group (aRR: p-value=0.1; aRD: p-value<0.001)
*Age at first positive SARS-CoV-2 RT-PCR test within University of Washington Medicine
†Conditions possibly associated with increased risk of severe COVID-19 per the United States Centers for Disease Control and Prevention [1], (all yes/no) based on electronic health record data; see Supplementary Table 1 for list of conditions
‡An indicator variable of COVID-19-associated hospitalization within University of Washington Medicine
§Adjusted for continuous age (years), sex assigned at birth (female/male), race and ethnicity (Hispanic or Latine, non-Hispanic American Indian or Alaska Native, non-Hispanic Asian, non-Hispanic Black, non-Hispanic Native Hawaiian or Pacific Islander, non-Hispanic White, not recorded), and health insurance status (public, private, uninsured, not recorded); total models also adjusted for age group (18-39, 40-64, 65+ years)
‖Difference in cumulative incidence per 1,000 SARS-CoV-2-positive persons over the 13-month study period

Supplementary Table 5. Risks, risk ratios, and risk differences of COVID-19-associated hospitalization by presence of any UHC among patients with a positive SARS-CoV-2 RT-PCR test, overall and by age group, University of Washington Medicine healthcare system, July 1, 2020-October 31, 2020

**Age group (years)***	**Any UHC†**	**Risk of COVID-19 hospitalization‡**		**Crude RR**		**Adjusted RR§**		**Crude RD‖**		**Adjusted RD§‖**	
		**n/N**	**%**	**RR**	**95% CI**	**aRR**	**95% CI**	**RD**	**95% CI**	**aRD**	**95% CI**
18-39	Yes	16 / 640	2.5	6.4	1.5, 27.7	4.9	1.2, 19.9	21	8, 34	13	0, 26
	No	2 / 511	0.4	*Referent*		*Referent*		*Referent*		*Referent*	
40-64	Yes	28 / 583	4.8	8.5	1.2, 61.9	7.0	0.9, 52.5	42	22, 63	36	15, 57
	No	1 / 176	0.6	*Referent*		*Referent*		*Referent*		*Referent*	
65+	Yes	21 / 237	8.9	3.2	0.4, 23.1	2.5	0.4, 16.6	61	-4, 126	55	-9, 119
	No	1 / 36	2.8	*Referent*		*Referent*		*Referent*		*Referent*	
Total	Yes	65 / 1,460	4.5	8.0	2.9, 22.0	4.8	1.8, 13.0	39	27, 51	23	11, 34
	No	4 / 723	0.6	*Referent*		*Referent*		*Referent*		*Referent*	

COVID-19=coronavirus disease 2019, UHC=underlying health condition, SARS-CoV-2=severe acute respiratory syndrome coronavirus 2, RT-PCR=reverse transcription polymerase chain reaction, (a)RR=(adjusted) risk ratio, (a)RD=(adjusted) risk difference, CI=confidence interval
RRs estimated by log-binomial regression using Huber-White estimates of the standard error; RDs estimated using a generalized linear model with a Gaussian distribution and identity link function, and using Huber-White estimates of the standard error
Analysis of variance used to test for interaction by age group (aRR: p-value=0.8; aRD: p-value=0.2)
*Age at first positive SARS-CoV-2 RT-PCR test within University of Washington Medicine
†Conditions possibly associated with increased risk of severe COVID-19 per the United States Centers for Disease Control and Prevention [1], (all yes/no) based on electronic health record data; see Supplementary Table 1 for list of conditions
‡An indicator variable of COVID-19-associated hospitalization within University of Washington Medicine
§Adjusted for continuous age (years), sex assigned at birth (female/male), race and ethnicity (Hispanic or Latine, non-Hispanic American Indian or Alaska Native, non-Hispanic Asian, non-Hispanic Black, non-Hispanic Native Hawaiian or Pacific Islander, non-Hispanic White, not recorded), and health insurance status (public, private, uninsured, not recorded); total models also adjusted for age group (18-39, 40-64, 65+ years)
‖Difference in cumulative incidence per 1,000 SARS-CoV-2-positive persons over the 13-month study period

Supplementary Table 6. Risks, risk ratios, and risk differences of COVID-19-associated hospitalization by presence of any UHC among patients with a positive SARS-CoV-2 RT-PCR test, overall and by age group, University of Washington Medicine healthcare system, February 29, 2020-March 13, 2021

Age group (years)*	Any UHC†	Risk of COVID-19 hospitalization‡		Crude RR		Adjusted RR§		Crude RD‖		Adjusted RD§‖	
		n/N	%	RR	95% CI	aRR	95% CI	RD	95% CI	aRD	95% CI
18-39	Yes	38 / 1,362	2.8	5.3	2.6, 10.5	4.2	2.1, 8.5	23	13, 32	14	4, 24
	No	10 / 1,887	0.5	*Referent*		*Referent*		*Referent*		*Referent*	
40-64	Yes	122 / 1,923	6.3	9.7	4.3, 21.9	7.8	3.5, 17.7	57	45, 69	48	36, 60
	No	6 / 917	0.7	*Referent*		*Referent*		*Referent*		*Referent*	
65+	Yes	143 / 1,133	12.6	2.9	1.6, 5.4	2.2	1.2, 4.1	83	50, 115	73	40, 106
	No	10 / 230	4.3	*Referent*		*Referent*		*Referent*		*Referent*	
Total	Yes	303 / 4,418	6.9	8.0	5.4, 11.9	4.2	2.8, 6.4	60	52, 68	34	26, 42
	No	26 / 3,034	0.9	*Referent*		*Referent*		*Referent*		*Referent*	

COVID-19=coronavirus disease 2019, UHC=underlying health condition, SARS-CoV-2=severe acute respiratory syndrome coronavirus 2, RT-PCR=reverse transcription polymerase chain reaction, (a)RR=(adjusted) risk ratio, (a)RD=(adjusted) risk difference, CI=confidence interval
RRs estimated by log-binomial regression using Huber-White estimates of the standard error; RDs estimated using a generalized linear model with a Gaussian distribution and identity link function, and using Huber-White estimates of the standard error
Analysis of variance used to test for interaction by age group (aRR: p-value=0.04; aRD: p-value<0.001)
*Age at first positive SARS-CoV-2 RT-PCR test within University of Washington Medicine
†Conditions conclusively associated with increased risk of severe COVID-19 per the United States Centers for Disease Control and Prevention [1], (all yes/no) based on electronic health record data; see Supplementary Table 1 for list of conditions
‡An indicator variable of COVID-19-associated hospitalization within University of Washington Medicine
§Adjusted for continuous age (years), sex assigned at birth (female/male), race and ethnicity (Hispanic or Latine, non-Hispanic American Indian or Alaska Native, non-Hispanic Asian, non-Hispanic Black, non-Hispanic Native Hawaiian or Pacific Islander, non-Hispanic White, not recorded), and health insurance status (public, private, uninsured, not recorded); total models also adjusted for age group (18-39, 40-64, 65+ years)
‖Difference in cumulative incidence per 1,000 SARS-CoV-2-positive persons over the 13-month study period

REFERENCES

1. COVID-19 - Underlying Medical Conditions. Centers for Disease Control and Prevention. 2022. https://www.cdc.gov/coronavirus/2019-ncov/science/science-briefs/underlying-evidence-table.html. Accessed 16 Mar 2022.

2. Littman AJ, Boyko EJ, McDonell MB, Fihn SD. Evaluation of a Weight Management Program for Veterans. Prev Chronic Dis. 2012;9:110267.
